# Energy recovery from syngas and pyrolysis wastewaters with anaerobic mixed cultures

**DOI:** 10.1186/s40643-024-00791-3

**Published:** 2024-07-27

**Authors:** Alberto Robazza, Anke Neumann

**Affiliations:** Institute of Process Engineering in Life Sciences 2: Electro Biotechnology, Karlsruhe Institute of Technology, KIT, 76131 Karlsruhe, Germany

**Keywords:** Pyrolysis wastewater, Phenolics, Nitrogen heterocycles, Open cultures, Volatile fatty acids, Sewage sludge, Polyethylene, Energy recovery, Carbon capture

## Abstract

The anaerobic digestion of aqueous condensate from fast pyrolysis is a promising technology for enhancing carbon and energy recovery from waste. Syngas, another pyrolysis product, could be integrated as a co-substrate to improve process efficiency. However, limited knowledge exists on the co-fermentation of pyrolysis syngas and aqueous condensate by anaerobic cultures and the effects of substrate toxicity. This work investigates the ability of mesophilic and thermophilic anaerobic mixed cultures to co-ferment syngas and the aqueous condensate from either sewage sludge or polyethylene plastics pyrolysis in semi-batch bottle fermentations. It identifies inhibitory concentrations for carboxydotrophic and methanogenic reactions, examines specific component removal and assesses energy recovery potential. The results show successful co-fermentation of syngas and aqueous condensate components like phenols and N-heterocycles. However, the characteristics and load of the aqueous condensates affected process performance and product formation. The toxicity, likely resulting from the synergistic effect of multiple toxicants, depended on the PACs’ composition. At 37 °C, concentrations of 15.6 g_COD_/g_VSS_ and 7.8 g_COD_/g_VSS_ of sewage sludge-derived aqueous condensate inhibited by 50% carboxydotrophic and methanogenic activity, respectively. At 55 °C, loads between 3.9 and 6.8 g_COD_/g_VSS_ inhibited by 50% both reactions. Polyethylene plastics condensate showed higher toxicity, with 2.8 g_COD_/g_VSS_ and 0.3 g_COD_/g_VSS_ at 37 °C decreasing carboxydotrophic and methanogenic rates by 50%. At 55 °C, 0.3 g_COD_/g_VSS_ inhibited by 50% CO uptake rates and methanogenesis. Increasing PAC loads reduced methane production and promoted short-chain carboxylates formation. The recalcitrant components in sewage sludge condensate hindered e-mol recovery, while plastics condensate showed high e-mol recoveries despite the stronger toxicity. Even with challenges posed by substrate toxicity and composition variations, the successful conversion of syngas and aqueous condensates highlights the potential of this technology in advancing carbon and energy recovery from anthropogenic waste streams.

**Graphical Abstract:**

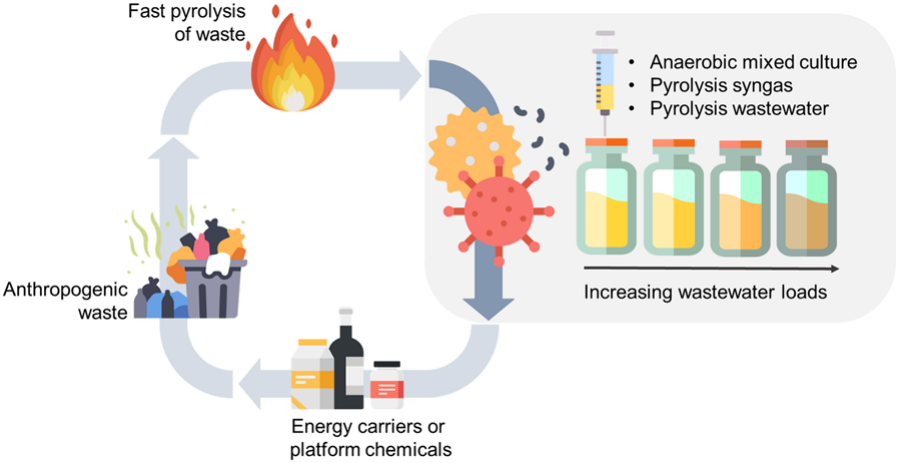

**Supplementary Information:**

The online version contains supplementary material available at 10.1186/s40643-024-00791-3.

## Introduction

Improper management of municipal sewage sludge (SS) and post-consumer plastics such as polyethylene (PE) poses risks to both human health and the environment. Common disposal methods, including landfilling, agricultural use of SS and incineration have some drawbacks. For example, landfilling can cause leachate pollution and greenhouse gas emissions, while agricultural applications of SS may elevate the concentrations of heavy metals in the soil. Incineration, on the other hand, produces noxious volatile compounds, requiring costly technologies to control pollutant emissions (Cies̈lik et al. 2015). Additionally, the recycling of post-consumer plastics is not entirely closed-loop, resulting in some waste persisting as micro plastics in the environment (Evode et al. 2021).

Considering the escalating volume of waste generated, a reassessment of available technologies is imperative to reduce environmental impacts whilst maximising energy and resources recovery from both wastes (Fonts et al. 2012). Thermochemical recycling technologies (such as hydrothermal conversion, gasification or pyrolysis) have the potential to address waste management challenges by reducing dependence on landfilling, minimizing solid residue volumes and mitigating greenhouse gas emissions and other harmful pollutants (Usman et al. 2019). Pyrolysis, for instance, yields products such as biochar, bio-oil, syngas (a mixture of CO, H_2_, CO_2_, CH_4_ and other alkanes and alkenes in smaller concentration) and a pyrolysis aqueous condensate (PAC) (Posmanik et al. 2017). In general, biochar, bio-oil and syngas are the target products of thermochemical processes, exhibiting heating values comparable to other fuels. The PAC, on the other hand, although accounting for about 20–30% of the wet mass of the original feed, is considered a by-product due to high water content and low calorific value, thereby lowering pyrolysis efficiency (Niebel et al. 2021).

The applications and handling of PAC are limited by its toxicity and complexity (Posmanik et al. 2017; Chen, et al. 2018). PAC can contain hundreds of compounds including organic acids, phenolics, amines, amides, guaiacols, furans, hydrocarbons and nitrogen heterocycles (Alvarez et al. 2016; Jaramillo-Arango et al. 2016). Many more remain poorly characterized posing challenges in assessing their specific inhibitory effects on microbial activity (Parku et al. 2023; Black et al. 2016). In addition, traces of bio-oil can be also contaminating the aqueous condensate (Chen, et al. 2018). In general, the composition of PAC is primarily determined by the type of feedstock. In the case of the PAC generated from the pyrolysis of sewage sludge (SS-PAC), for instance, the concentration of different constituents depends upon the levels proteins, lipids and lignin of the original waste (Wang et al. 2021). Phenolic compounds can arise from the volatilization of polysaccharides and proteins, aromatic hydrocarbons stem from the decarboxylation of fatty acids within the lipid fraction, while ammonium and N-heterocycles are some products of protein decomposition (Chanaka Udayanga et al. 2019). Carbocyclic acids, phenolics and hydrocarbons can be found also in the PAC resulting from the pyrolysis of mixed polyethylene plastics (PE-PAC) and originate from organic impurities of the waste (Zeller et al. 2021). The type and concentration of PAC components determine the chemical properties and the toxicity of the solution (Seyedi et al. 2019). SS-PAC, for instance, exhibits basic pH, whereas PE-PAC is acidic. Despite both PACs may contain similar concentrations of carboxylic acids, the pH disparity may depend upon the presence of high concentration of ammonium and nitrogen aromatics in SS-PAC (Usman et al. 2019; Funke et al. 2017). The integration of thermochemical and biological processes into a unified platform represents a technology that could improve the energy and resource recovery from pyrolysis by-products and enhance the economic sustainability of the process on an industrial scale (Watson et al. 2020; Arnold et al. 2019). Previous attempts to valorise various PACs in single-culture fermentations encountered challenges that required extensive detoxification through physicochemical pre-treatments (Liang et al. 2013). Anaerobic digestion, on the other hand, stands out as a viable technology for recovering energy from PAC components such as carboxylic acids, anhydrous sugars and aromatic compounds, among others, fermenting them into biogas. Various factors such as the microbial composition of the inoculum, concentration of refractory and toxic compounds, the molecular weight of PAC components, PAC loading rate and process operating conditions affect process performances (Chen et al. 2020; García Rea, et al. 2022). The addition of suitable amendments such as biochar has been proven successful to mitigate the toxicity of PAC compounds on methanogenesis (Watson et al. 2020; Torri and Fabbri 2014). Alternatively, since syntrophic and acidogenic microorganisms play a crucial role in the degradation of PAC components, their enrichment or bio-augmentation could serve as a viable strategy to enhance PAC degradation and overall process performance (Watson et al. 2020; Küçükaǧa et al. 2022; Zhou et al. 2019).

Currently, coal gasification and steam reforming of natural gas stand as the primary methods for syngas production, albeit generating substantial green-house gas emissions. Conversely, the steel-manufacturing industry and thermochemical conversion of waste offer alternative sources of syngas characterized by lower greenhouse gas emissions (Bachmann et al. 2023). However, with ongoing technological advancements in electrolysis for H_2_ production, there is potential for steel-manufacturing industries to achieve full decarbonisation of their processes (Arens et al. 2017), leaving thermochemical conversion of waste as one of the remaining sources of syngas. The biological conversion of syngas into biofuels or other valuable commodity chemicals is emerging as a key biotechnological tool for establishing a circular bioeconomy (Fuchs et al. 2023). Syngas fermentation has been explored for the production of biopolymers, single cell proteins, medium-chain carboxylates and other valuable compounds, showcasing its potential and versatility (Khalid et al. 2023). Research on anaerobic syngas fermentation centers predominantly on acetogens such as *Clostridium ljungdahlii*, *Clostridium autoethanogenum*, *Acetobacterium woodii* and *Moorella thermoacetica*, which employ the Wood-Ljungdahl pathway to fix CO and H_2_/CO_2_, producing acetyl-CoA as the central intermediate (Perret et al. 2022). Alternatively, syngas fermentation with mixed anaerobic cultures has shown potential for CH_4_ or carboxylates production. Beyond acetogens, other microbes capable of metabolizing syngas within anaerobic cultures include certain hydrogenogenic bacteria, methanogenic archaea and sulfate-reducing bacteria (Navarro et al. 2016). While CH_4_ is often the primary metabolite of mixed culture syngas fermentation (Liu et al. 2018; Kleerebezem and Loosdrecht 2007), innovative research has developed processes for producing short and medium-chain carboxylates (Baleeiro et al. 2019; Angenent et al. 2016). Incorporating syngas as a substrate during the anaerobic fermentations of organic waste could provide extra electron donors to the process, enhancing energy recovery while minimizing gaseous emissions (Angenent et al. 2016).

In this context, despite both syngas and PAC originating from the pyrolysis process, knowledge regarding their co-fermentation remains limited. Only few works have focussed on the co-fermentation of syngas and lignocellulose PAC, demonstrating the capability of anaerobic mixed cultures to concurrently perform syngas sequestration and degradation of pyrolysis-derived organic components for biogas and short-chain carboxylates (SCCs) production (Küçükaǧa et al. 2022; Robazza et al. 2022). One study explored the co-fermentation of syngas, PAC and glucose for carboxylates production in a continuous biochar-packed reactor (Küçükaǧa et al. 2022), while another one demonstrated the potential of a two-stage process, aerobic to anaerobic, for converting the carboxylates produced from syngas and PAC co-fermentation in the initial stage into L-malate in the second stage (Robazza et al. 2022). However, additional research dedicated to syngas and PACs co-fermentation is necessary to broaden the understanding of this technology and its potential for integrating thermochemical waste conversion with biological processes to enhance energy recovery from waste.

The objective of this work is to investigate the effects of the PAC composition on the metabolism of mesophilic and thermophilic anaerobic mixed cultures during the co-fermentation of syngas and either SS-PAC or PE-PAC. By conducting tests at increasing PAC loadings in 250 mL bottles, the study elucidates kinetic inhibitions, metabolic shifts and the energetics of the process in correlation to the specific PAC cell load. The results of this work will contribute to a deeper understanding of anaerobic mixed cultures' potential to perform syngas sequestration and wastewater detoxification under diverse toxicants pressure. Bridging this knowledge gap is essential for unlocking the full potential of these waste streams in achieving a more sustainable and efficient waste conversion and energy recovery.

## Materials and methods

### Inoculum and PACs

The inoculum was collected at an anaerobic digester treating cow-manure as described in another work (Robazza et al. 2022). The total suspended solids (TSS) and the volatile suspended solids (VSS) of the inoculum were 41.1 ± 0.8 g/L and 18.9 ± 0.8 g/L, respectively, and were determined following the procedure described in the Method 1684 (Telliard 2001).

Both SS-PAC and PE-PAC were provided by the Institute of Technical Chemistry (ITC) at the Karlsruhe Institute of Technology, Campus Nord (Karlsruhe, Germany). The pyrolysis experiments were carried out using a pilot scale screw reactor system (Tomasi Morgano et al. 2018). The SS-PAC used in this work was a 1:1 mixture of the PACs generated during the pyrolysis of a dried and non-dried sewage sludge. The pyrolysis process was non-catalyzed and performed at 500 °C, with a residence time of 15 min and a loading rate of 1.2 kg/h. SS-PAC had a pH of 9.6, a chemical oxygen demand (COD) of 185 g/L, a total organic carbon of 109 g/L, a total nitrogen concentration of 84.2 g/L and contained 92.6 ± 3.5 g/L of NH_4_^+^, 9.9 ± 0.02 g/L acetate, 3.7 ± 0.05 g/L propionate and 1.8 ± 0.01 g/L butyrate. Similarly, the PE-PAC was a 1:1 mixture of two PACs produced during the pyrolysis of heavy weight and light weight mixed PE plastics. The pyrolysis was run at 450 °C with a residence time of 30 min and a waste loading rate of 1 kg/h using zeolite as catalyst. PE-PAC had a pH of 1.8, a COD of 89.9 g/L, a total organic carbon of 30 g/L and contained 10.96 g/L of total nitrogen, 7.82 + 0.01 g/L of NH_4_^+^, 14.3 ± 0.3 g/L acetate, 2.7 ± 0.01 g/L propionate and 0.6 ± 0.1 g/L of butyrate. The mass balances of the pyrolysis processes and a more detailed composition of the PACs are available in the Supplementary Information (Additional file 1, Table S1, Table S2 and Table S3).

### Fermentation

The bottle fermentations were conducted over a 10-day period, in triplicate, in 250 mL serum bottles with a 50 mL active volume. The fermentation medium comprised 5% v/v BA medium (Robazza et al. 2022), a variable PAC amount based on the experimental design and deionized water as required to meet 45 mL. Either 4 M NaOH or 4 M H_3_PO_4_ was employed to adjust the pH post PACs addition. To account for the significant pH disparity between the two PACs and the necessity to minimize salts addition, the pH for SS-PAC bottles was set to 7.2, while PE-PAC bottles were set at 6.6. SS-PAC loading for mesophilic and thermophilic co-fermentations ranged from 2 to 24% v/v with 2% increments, while PE-PAC concentrations ranged from 0.5 to 6% v/v with 0.5% increments. The PACs loading during co-fermentation experiments were designed to achieve at least 90% methanation inhibition compared to controls under either mesophilic or thermophilic conditions.

After 24 h of anaerobization in an anaerobic tent (5% H_2_ in N_2_), the bottles were inoculated with 10% v/v anaerobic sludge (resulting in 1.89 g_VSS_/L at inoculation) and were then sealed with butyl rubber stoppers and aluminium rings. Subsequently, each triplicate underwent syngas flushing (6 kPa H_2_, 21 kPa CO, 26 kPa CO_2_, and N_2_ at 1 L/min) and pressurization at room temperature to a final pressure of 210 kPa_abs_. The gas flow was regulated by high precision mass flow controllers (Vögtlin, Muttenz, Switzerland) and bottle pressure was monitored using a precision pressure indicator GMH 3100 Series (Greisinger, Mainz, Germany). Incubation occurred at 37 °or 55 °C and 210 rpm in two Thermotron shaker incubators (Infors, Bottmingen, Switzerland).

### Analytical methods

Daily pressure analysis and gas sampling were performed immediately after removal from the incubators. Three millilitres of the gas phase were sampled and analysed for the determination of the molar concentration of CO, CO_2_, H_2_, CH_4_, H_2_S and N_2_ using an Inficon 3000 Micro GC System with a Thermal Conductivity Detector (TCD) equipped with a CP-Molsieve 5 Å column and a PoraPLOT Q column at 80 °C using argon and helium as carrier gases, respectively. Every second day, 1 mL of the fermentation broth was sampled, centrifuged, filtered and stored at – 20 °C for later analytics. The concentrations of formate, acetate, propionate, *n*-butyrate and of some selected PAC compounds (phenol, guaiacol and *o*-,*m*-, *p-*cresol) were measured by a high-performance liquid chromatography (HPLC) (Agilent 1100 Series, Agilent, Waldbronn, Germany) at 55 °C with a Rezex ROA organic acid H^+^ (8%) column (300 × 7.8 mm, 8 µm; Phenomenex, Aschaffenburg, Germany) and a Rezex ROA organic acid H + (8%) guard column (50 by 7.8 mm). The mobile phase was 5 mM H_2_SO_4_ at a flow of 0.6 mL/min. Benzene, triacetoneamine, benzonitrile and pyridine were quantified by means of high-performance liquid chromatography with an Agilent 1100 Series (Agilent, Waldbronn, Germany) at 40 °C with a Kinetex 250 × 4.6 mm, 2.6 µm EVO C18 column (Phenomenex, Aschaffenburg, Germany) and a SecurityGuard 4 × 3.0 mm C18 guard column (Phenomenex, Aschaffenburg, Germany). The eluents were composed by a mixture of 0.1% v/v ethanolamine in H_2_O and 0.1% v/v ethanolamine in acetonitrile flowing at 1.1 mL/min. The elution profile was: 0.5% at 0 min; 0.5% at 5 min; 38.5% at 18 min; 90% at 20 min; 90% at 23 min; 0.5% at 25 min. Sulphate and nitrate concentrations were measured by ion chromatography with a Metrohm 930 Compact IC Flex (Metrohm, Filderstadt, Germany) equipped with a Metrosep A Supp 5–150/4.0. A solution of 1 mM of NaHCO_3_ and 3.2 mM of Na_2_CO_3_ at 0.8 mL/min was used as eluent while 500 mM H_2_SO_4_ was used as suppressor. Photometrical quantification of the total ammonia nitrogen (TAN) concentrations were performed with a Spectroquant kit 114752 (Merck KGaA, Darmstadt, Germany). Free ammonia nitrogen (FAN) represents the un-ionized part of TAN and depends primarily upon pH and temperature (Eq. 1 and 2) (Wang, et al. 2022).

1 $${\text{C}}_{\text{FAN}}=\frac{{\text{C}}_{\text{TAN}}}{1+{10}^{({\text{pK}}_{\text{a}}-\text{pH})}}\left[\text{mM}\right]$$

2 $${\text{pK}}_{\text{a}}=0.09018+\frac{2792.92}{\text{T}+273.15}$$

pK_a_ is the dissociation constant for ammonium ion, 8.892 at 37 °C while 8.408 at 55 °C. T is the temperature, °C. The molar amounts of each gas specimen (CO, H_2_, CO_2_, CH_4_) were determined at each sampling point using the ideal gas law, taking into account both pressure loss and air contamination during sampling. The differences in molar amounts between successive samplings were cumulated to yield the total quantity of gas produced or consumed (Eq. 3).

3 $${\text{n}}_{\text{gas},\text{ i}}=\sum_{\text{t}=0}^{\text{j}}\frac{{\text{p}}_{\text{j}}{*\text{V}}_{\text{j}}}{\text{R}*\text{T}}[\text{mmol}]$$

where ${n}_{\text{gas},\text{ i}}$ is the cumulative absolute consumption/production of a gas specimen throughout the total fermentation time; ${\text{p}}_{\text{j}}$ is the pressure of the bottle’s head space at sampling time corrected to account for pressure loss by sampling; ${\text{V}}_{\text{j}}$ is the bottle’s head space volume; $\text{R}$ is the gas constant; $\text{T}$ is the incubation temperature; $\text{j}$ is the number of samples.

The amount of gases and the difference in metabolites levels (Eq. 4) (formate, acetate, propionate and butyrate) between initial and final samples were multiplied by the corresponding electron equivalents (eeq) and divided by the total fermentation time and initial broth volume to calculate space–time consumption/production rates of electron moles (e-mol).

4 $${\text{q}}_{\text{e}-\text{mol},\text{ i}}=\frac{{\text{n}}_{\text{i}}*{\text{eeq}}_{\text{i}}}{{\text{V}}_{\text{Start}}*\text{t}}[\text{e}-\text{mM}/\text{d}]$$

where ${n}_{\text{i}}$ is absolute amount of each metabolite produced or consumed during the total fermentation time; ${eeq}_{i}$ is the amount of the substance $i$ which releases 1 e-mol during complete oxidation; ${V}_{Start}$ is the active volume at the start of the fermentation; $t$ is the total fermentation time.

The e-mol recoveries were calculated as described in Eq. 5.

5 $$\text{e}-\text{mol recovery}=\frac{\sum {\text{n}}_{\text{i}}*{\text{eeq}}_{\text{i}}}{{\text{n}}_{\text{Syngas},\text{ fed}}*{\text{eeq}}_{\text{syngas}}+{8*\text{gCOD}}_{\text{PAC},\text{fed}}}*100[\%]$$

The e-mol_PAC_ were calculated assuming that 8 gCOD is equal to one e-mol. Considering that the COD is the oxygen required to completely oxidize the carbonaceous fraction of organic compounds and that 1 eeq. is released upon complete oxidation of carbonaceous compounds, then from the half reaction RS1, it can assumed that 1/4 mol of O_2_ (8 g) would be consumed in accepting the 1 e-mol.

$$1/2{\text{H}}_{2}\text{O}= 1/4{\text{O}}_{2}+{\text{H}}^{+}+{\text{e}}^{-}$$ R.1

The e-mol balances and e-mol recoveries from PAC were calculated as outlined in the following equations.

6 $$\text{e}-\text{balance }=\frac{\sum {\text{n}}_{\text{i}}*{\text{eeq}}_{\text{i}}}{{\text{n}}_{\text{CO}}*{\text{eeq}}_{\text{CO}}+{\text{n}}_{{\text{H}}_{2}}*{\text{eeq}}_{{\text{H}}_{2}}}*100[\%]$$

7 $$\text{e}-\text{mol recovery from PAC }=\frac{\sum {\text{n}}_{\text{i}}*{\text{eeq}}_{\text{i}}-{\text{n}}_{\text{Syngas},\text{ fixed}}*{\text{eeq}}_{\text{syngas}}}{{8*\text{g}}_{\text{PAC},\text{fed}}}\times 100[\%]$$

where ${n}_{\text{i}}$ is absolute amount of each metabolite produced or consumed during the total fermentation time; ${eeq}_{i}$ is the amount of the substance $i$ which releases 1 e-mol during complete oxidation of any organic carbonaceous compound*.* Table S5 in the Additional file 1 reports the conversion factors used for electron balancing. All calculations were conducted individually for each bottle and the results were subsequently averaged across the replicates (*n* = 3).

## Results

This chapter illustrates the potential for carbon and e-mol recovery from syngas and two distinct PACs through anaerobic mixed culture fermentations. These waste streams result from the pyrolysis of sewage sludge and a combination of high and low density PE plastics. The analysis focuses on the space–time rates of syngas conversion into short-chain carboxylates at increasing PACs loading, evaluates the energy recoveries from both syngas and PACs and assesses the efficiency of removing some selected PAC components.

### Syngas and sewage sludge PAC co-fermentation

The results from the mesophilic syngas and SS-PAC co-fermentation experiments (M-SS-PAC) will be presented first, followed by the thermophilic syngas and SS-PAC co-fermentation experiments (T-SS-PAC). In general, at both temperatures conditions, the cultures exhibited the ability to perform concurrent syngas sequestration and carbon-energy recovery from SS-PAC.

**Fig. 1:**
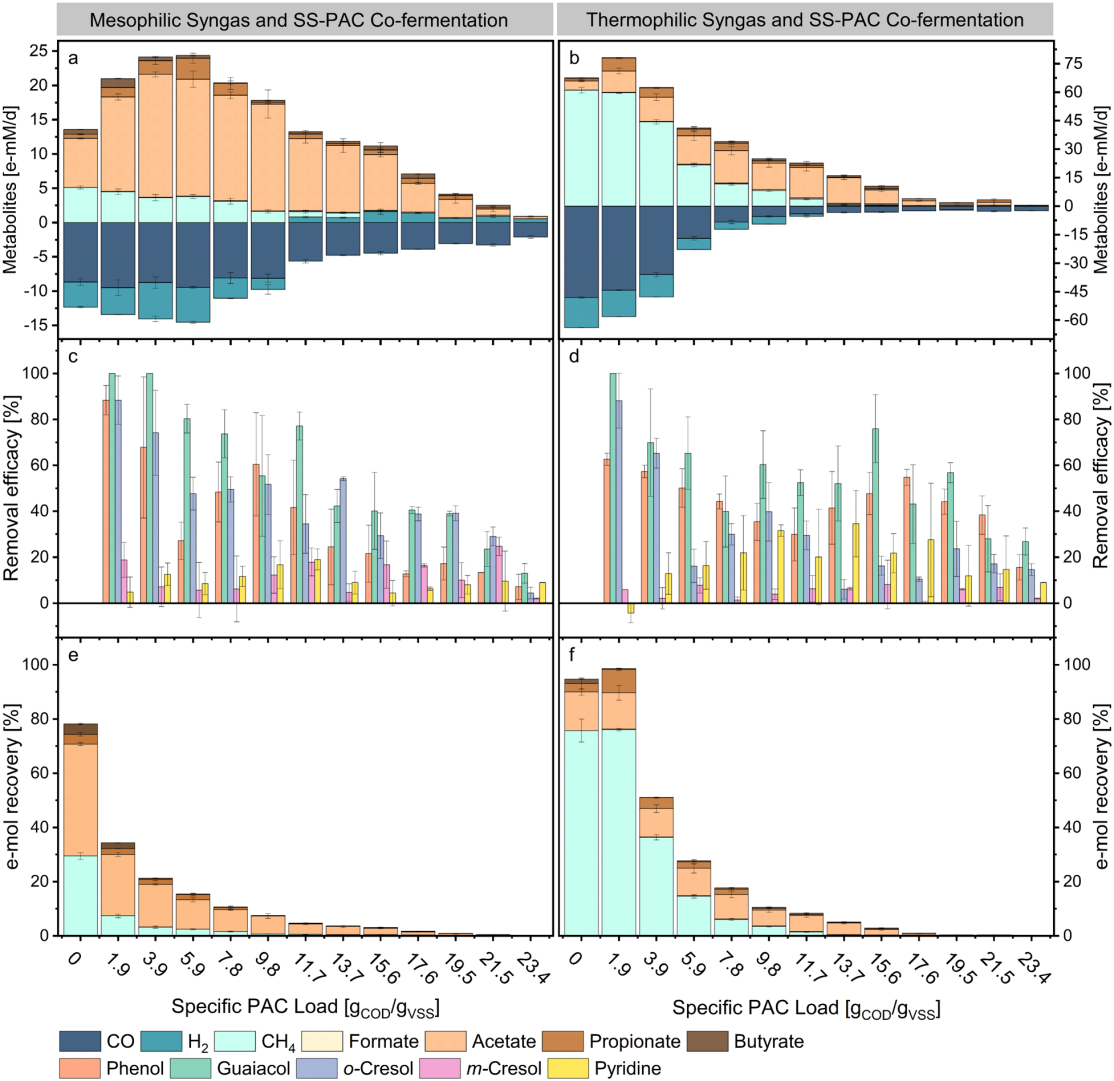
Effects of increasing SS-PAC loading on the metabolism of mesophilic (**a, c, e**) and thermophilic (**b, d, f**) mixed cultures. The graphs **a** and **b** show the kinetic rates of CO, H_2_, CH_4_, formate, acetate, propionate and butyrate for each load of SS-PAC. The colours highlight increasing e-equivalence of the metabolites detected. Negative values indicate consumption. The bar-graphs **c** and **d** show the removal efficacies of some SS-PAC compounds clustered for each load of SS-PAC. The bar-graphs **e** and **f** illustrate the e-mol recoveries from substrates (syngas fed and PAC) into products (methane, formate, acetate, propionate, butyrate). Error bars represent standard deviation among replicates (*n* = 3)

At 37 °C, CO consumption rates showed no significant inhibition up to the SS-PAC loading of 9.8 g_COD_/g_VSS_ but decreased linearly at greater loads (Fig. 1a). At the SS-PAC load of 23.4 g_COD_/g_VSS_, carboxydotrophic rates were 2.1 ± 0.2 e-mM_CO_/d, marking a four-fold decrease from control experiments. Methanation, on the other hand, was inhibited at lower specific SS-PAC loadings: a 1.9 g_COD_/g_VSS_ SS-PAC loading caused a 12% inhibition of methanogenic rates compared to the control experiments, while at 11.7 g_COD_/g_VSS_ the methanation was reduced by over 90%. Corresponding initial FAN and TAN concentrations were 13.36 ± 0.3 mM (226.6 mg/L) and 629 ± 6.7 mM (11,342 mg/L), respectively. Initial concentrations of FAN and TAN in the other broths of M-SS-PAC experiments are available in the Supplementary Information (Additional file 1, Figure S1a). The inhibitory concentration causing 50% inhibition (IC50) of SS-PAC was determined to be 15.6 g_COD_/g_VSS_ for carboxydotrophic activity and around 7.8 g_COD_/g_VSS_ for methanogenesis. Hydrogenotrophic activity was detected up to SS-PAC loadings of 9.8 g_COD_/g_VSS_, coincidently to methane production. For higher SS-PAC loadings, the highest hydrogen production rate was 1.6 ± 0.38 e-mM_H2_/d at 15.6 g_COD_/g_VSS_. Acetate constituted approximately 58% of the total e-mol sequestered from syngas during control experiments alongside traces of propanol and butyrate. Similarly, acetate, propionate and butyrate were the primary metabolites accumulating in the fermentation broth during syngas and SS-PAC degradation. The final pH for each condition are available in the Additional file 1 (Figure S4a). The e-mol recovery from syngas and SS-PAC (Fig. 1e) peaked to 34.3 ± 0.7% at 1.9 g_COD_/g_VSS_. Similarly, the highest e-mol recovery from exclusively SS-PAC was 17.3 ± 2.9% at 1.9 g_COD_/g_VSS_ (Additional file 1, Figure S3a). Both recoveries decreased towards zero at increasing loadings of SS-PAC. The removal efficiency of selected SS-PAC components supports the degradation of phenolics, pyridine and possibly other SS-PAC constituents during the co-fermentation (Fig. 1c). All SS-PAC components exhibited a positive removal efficacy, albeit with a diminishing trend as SS-PAC loadings increased.

During the thermophilic control experiments, CO oxidation occurred at a rate of 48.1 ± 0.4 e-mM_CO_/d, 6 times greater than the mesophilic control experiments (Fig. 1b). However, it sharply declined concomitant with the incremental addition of SS-PAC. Methanogenic rates mirrored to those of syngas uptake. Traces of carboxydotrophic activity (2.4 ± 0.2 e-mM_CO_/d, 95% inhibition) were detectable up to 23.4 g_COD_/g_VSS_. Methanation was completely arrested at 11.7 g_COD_/g_VSS_. Corresponding initial FAN and TAN concentrations were 41.7 ± 0.6 mM (709.8 mg/L) and 674 ± 10.3 mM (12,169 mg/L), respectively. Initial concentrations of FAN and TAN in the other broths of T-SS-PAC experiments are available in the Supplementary Information (Additional file 1, Figure S1b). The influence of increasing SS-PAC loadings at 55 °C had a more pronounced impact on carboxydotrophic rates compared to mesophilic experiments. The IC50 was determined to fall between 3.9 and 6.8 g_COD_/g_VSS_ SS-PAC loadings for both carboxydotrophic and methanogenic reactions. No significant hydrogen production was detected for SS-PAC loading higher than 17.6 g_COD_/g_VSS_. In control experiments, acetate and propionate constituted over 95% of the metabolites in the medium, with selectivities of approximately 85% and 10%, respectively.

Similarly, acetate and propionate were the primary metabolites in T-SS-PAC experiments. However, with higher SS-PAC amounts, increasing levels of butyrate were observed, reaching a peak productivity of 1.31 ± 0.2 e-mM_Butyrate_/d at 11.7 g_COD_/g_VSS_. Methane inhibition coincided to formate accumulation in the medium at a maximum rate of 0.67 ± 0.01 e-mM_Formate_/d at 15.6 g_COD_/g_VSS_. Thermophilic control experiments produced about 25% fewer e-mol_SCCs_ than mesophilic controls. Conversely, between loads of 1.9 and 15.6 g_COD_/g_VSS_, T-SS-PAC experiments produced 17% more e-mol_SCCs_ than M-SS-PAC experiments, peaking at approximately 60% at 11.7 g_COD_/g_VSS_. The average final pH for each SS-PAC loading is provided in the Supplementary Information (Additional file 1, Figure S2b). Fermentations with low SS-PAC loadings (between 0 and 11.7 g_COD_/g_VSS_) resulted in a final pH lower than that recorded at the time of inoculation. Conversely, as SS-PAC loadings increased, the final pH exceeded the initial pH at inoculation. During the thermophilic co-fermentation of syngas and SS-PAC, phenolics and pyridine were removed from the fermentation broths (Fig. 1d). The removal of *m-*cresol was very low.

The higher syngas uptake rates at thermophilic temperatures than those observed in mesophilic experiments contributed to higher e-mol recoveries from both syngas and SS-PAC. For instance, the peak e-mol recovery of 98.6 ± 6.2% at a loading of 1.9 g_COD_/g_VSS_ was approximately three times higher than the corresponding mesophilic experiment. Averaging across all conditions, T-SS-PAC fermentations recovered 1.4-fold more e-mols than the M-SS-PAC experiments (Fig. 1f). Similarly, the thermophilic e-mol recovery from SS-PAC alone was, on average, about 50% higher than mesophilic e-mol recoveries (Additional file 1, Figure S3b).

**Fig. 2:**
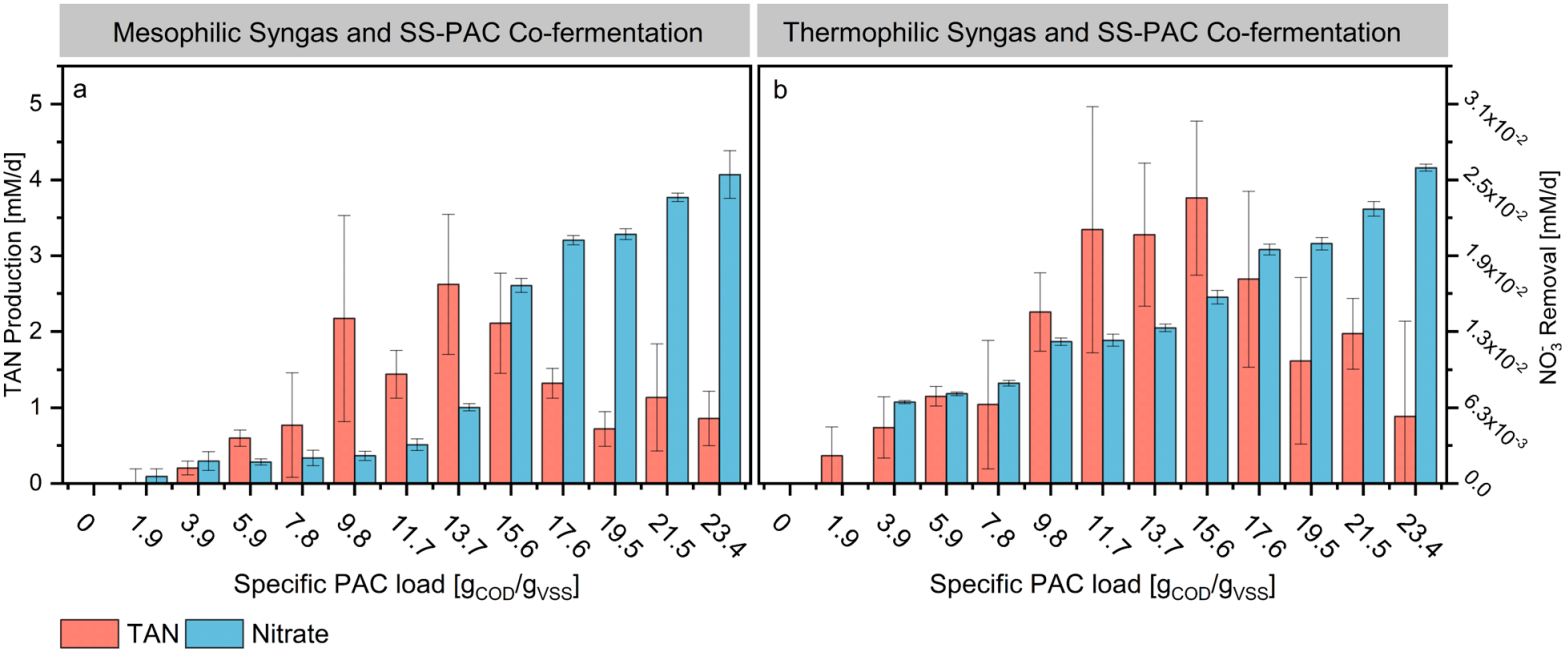
Total ammonium nitrogen (TAN) production rates and nitrate removal rates for mesophilic (**a**) and thermophilic (**b**) SS-PAC experiments. TAN production rates are red while nitrate removal rates are blue. The left Y-axis of graph **a** represents the scale for both M-SS-PAC and T-SS-PAC experiments. The right Y-axis of graph **b** represents the scale for both M-SS-PAC and T-SS-PAC experiments. Error bars represent standard deviation among replicates (*n* = 3)

When evaluating the e-mol balance between products (SCCs, methane and hydrogen) in comparison to syngas (excluding PAC), recoveries higher than 100% were consistently observed between SS-PAC loadings of 1.9 and 19.5 g_COD_/g_VSS_ (Additional file 1, Figure S4). This trend corroborates the fact that some products originate from the degradation of PAC components into SCCs. However, in both M-SS-PAC and T-SS-PAC and at specific SS-PAC loadings higher than 19.5 g_COD_/g_VSS_, the e-mol recoveries decreased below 100%. Mesophilic and thermophilic peak TAN production rates were 2.6 ± 0.9 mM/d and 3.8 ± 0.6 mM/d at 13.7 and 15.6 g_COD_/g_VSS_, respectively (Fig. 2a and b). At the same SS-PAC loadings, SCCs production rates were 10.5 ± 1.2 e-mM_SCCs_/d and 10.1 ± 1.6 e-mM_SCCs_/d. Nitrate was completely depleted in all of the fermentation media from both experimental sets. Sulphate, on the other hand, exhibited no significant changes between initial and final samples.

### Syngas and polyethylene plastics PAC co-fermentation

The following results illustrate the biomethanation and e-mol recovery capabilities of syngas and PE-PAC via anaerobic fermentations. The results from the mesophilic syngas and PE-PAC co-fermentation experiments (M-PE-PAC) will be presented first, followed by the thermophilic syngas and PE-PAC co-fermentation experiments (T-PE-PAC). In general, PE-PAC showed significantly higher toxicity to syngas conversion rates for both mesophilic and thermophilic mixed cultures compared to SS-PAC.

**Fig. 3:**
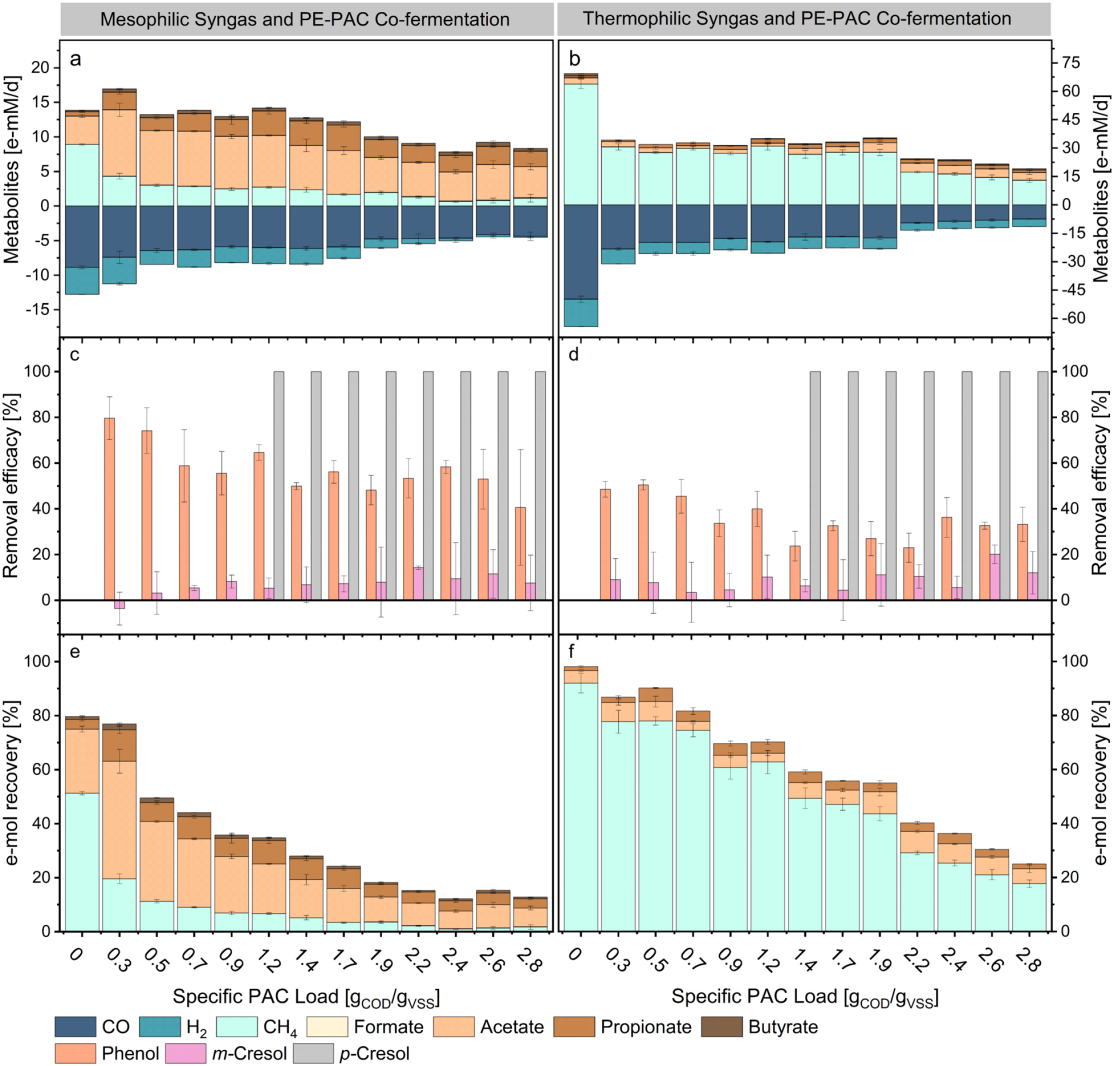
Effects of increasing SS-PAC loading on the metabolism of mesophilic (**a, c, e**) and thermophilic (**b, d, f**) mixed cultures. The graphs **a** and **b** show the kinetic rates of CO, H_2_, CH_4_, formate, acetate, propionate and butyrate for each load of PE-PAC. The colours highlight increasing e-equivalence of the metabolites detected. Negative values indicate consumption. The bar-graphs **c** and **d** show the removal efficacies of some PE-PAC compounds clustered for each load of PE-PAC. The bar-graphs **e** and **f** illustrate the e-mol recoveries from substrates (syngas fed and PAC) into products (methane, formate, acetate, propionate and butyrate). Error bars represent standard deviation among replicates (*n* = 3)

At 37 °C, CO uptake rates exhibited a linear decrease with increasing loadings of PE-PAC (Fig. 3a). In control experiments, carboxydotrophic activity measured 8.9 ± 0.2 e-mM_CO_/d but decreased to 4.4 ± 0.6 e-mM_CO_/d at a PE-PAC specific loading of 2.8 g_COD_/g_VSS_. Methanation rates, on the other hand, experienced a substantial reduction of approximately 90%, decreasing to 1.1 ± 0.6 e-mM_CH4_/d at 2.8 g_COD_/g_VSS_ PE-PAC compared to the 8.9 ± 0.1 e-mM_CH4_/d observed in control experiments. The PE-PAC loading causing 50% reduction of carboxydotrophic activity was 2.8 g_COD_/g_VSS_, whilst the IC50 for methanogenesis was 0.3 g_COD_/g_VSS_. Hydrogen consumption was detected in all conditions, with hydrogenotrophic rates decreasing linearly, paralleling the decline in methanogenic rates.

In mesophilic control fermentations, acetate and propionate constituted approximately 100% of the SCCs, with production rates of 4.1 ± 0.2 e-mM_Acetate_/d and 0.6 ± 0.1 e-mM_Propionate_/d, respectively. Traces of butyrate were also detected. Co-fermentation of syngas and PE-PAC enhanced acetate, propionate and butyrate productivities, reaching a peak cumulative value of 11.4 ± 0.8 e-mM_SCCs_/d at 1.2 g_COD_/g_VSS_. Acetate selectivity progressively decreased in favour of propionate but always remained above 60%. Propionate selectivity, on the other hand, increased from about 12% of the control experiments to about 35% at 1.7 g_COD_/g_VSS_. Formate production was detected concomitantly to reducing methanation rates. When considering PE-PAC components degradation, phenol was consistently removed from the fermentation media (Fig. 3c). Between loadings of 0.3 to 0.9 g_COD_/g_VSS_, the initial concentrations of *p-*cresol were below detection limit. At higher PE-PAC concentrations, *p*-cresol was completely removed from the fermentation broths. Similar to the removals obtained for SS-PAC, *m*-cresol showed minimal removal during the M-PE-PAC tests.

The e-mol recovery for mesophilic control experiments was 79.2 ± 2.4% (Fig. 3e). During syngas and PE-PAC co-fermentation, the e-mol recovery decreased linearly at increasing PE-PAC loadings. At a loading of 0.3 g_COD_/g_VSS_, the e-mol recovery from syngas and PAC was at its highest (76.9 ± 2.4%), while nearly 100% recovery was observed from PAC alone. Higher PE-PAC loadings hindered e-mol recovery, lowering it to 11.5 ± 0.2% at 2.8 g_COD_/g_VSS_.

At 55 °C, even the smallest PE-PAC loading of 0.3 g_COD_/g_VSS_, caused a 50% reduction in CO uptake rates. Carboxydotrophic rates remained above 17 e-mM_CO_/d (a 66% reduction compared to thermophilic controls) up to 1.9 g_COD_/g_VSS_. However, higher PE-PAC loadings led to a drop of CO uptake rates a drop to 7.5 ± 0.1 e-mM_CO_/d at 2.8 g_COD_/g_VSS_ (about 80% inhibition). Methane was the primary metabolite, regardless of the PE-PAC load (Additional file 1, Figure S5a). Similar to the T-SS-PAC tests, methanogenic rates were limited by CO oxidation kinetics and exogenous H_2_ availability, resulting in an identical IC50 of 0.3 g_COD_/g_VSS_ for methanogenesis.

In the control experiments, acetate, propionate and butyrate selectivities were about 59, 19 and 21%, respectively. Low PE-PAC loading favoured higher propionate ratios at the expenses of acetate but increasing PE-PAC loadings led to higher acetate and butyrate ratios. Acetate production rates increased steadily from 3.2 ± 0.2 e-mM_Acetate_/d of the control experiments to 5.1 ± 0.9 e-mM_Acetate_/d at 1.9 g_COD_/g_VSS_. Higher PE-PAC loadings, on the other hand, progressively lowered the acetate productivity to 4.1 ± 0.5 e-mM_Acetate_/d at 2.8 g_COD_/g_VSS_. Propionate productivity followed a trend similar to the one of acetate with decreasing rates after peaking to 2.45 ± 0.1 e-mM_Propionate_/d at 2.4 g_COD_/g_VSS_. Traces of butyrate production were detected at high PE-PAC loadings with the highest rate of 0.7 ± 0.03 e-mM_Butyrate_/d at 2.8 g_COD_/g_VSS_. No formate production was detected. Phenol showed stable removals, throughout all conditions (Fig. 3d). Similarly to M-PE-PAC tests, between loadings of 0.3 to 1.2 g_COD_/g_VSS_, initial *p-*cresol concentrations were too low to be detected. At higher PE-PAC loadings, *p-*cresol was completely removed from the fermentation broths. No consistent removal of *m*-cresol was detected.

High PE-PAC loadings resulted in the accumulation of acetate, propionate and butyrate in the fermentation medium possibly resulting from the degradation of PE-PAC compounds (Additional file 1, Figure S5b). Thermophilic temperatures improved energy recovery from both syngas and PE-PAC exhibiting e-mol recoveries on average two times higher than the M-PE-PAC experiments. The highest e-mol recoveries from syngas and PE-PAC were 99.8%, 86.8% and 90.2%, recorded at low PE-PAC loadings ranging between 0 and 0.5 g_COD_/g_VSS_. Higher PE-PAC loadings progressively lowered the e-mol recovery to about 25% at the PE-PAC loading of 2.8 g_COD_/g_VSS_. Similarly, the thermophilic mixed cultures recovered 72.6% of the e-mol of PE-PAC at 0.3 g_COD_/g_VSS_ but recoveries decreased to about 26% at PE-PAC loadings of 2.8 g_COD_/g_VSS_ (Additional file 1, Figure S7b).

## Discussion

### PACs inhibition on anaerobic mixed culture metabolism

Phenolics, N-heterocycles, TAN and FAN are among the components of SS-PAC that may have contributed to the inhibition of methanogenic and carboxydotrophic activity. A study examining the methane production during the anaerobic digestion of a PAC (with COD > 200 g/L and approximately 63 g_TAN_/L), derived from a non-catalyzed pyrolysis of sewage sludge, reported severe methanogenesis inhibition at PAC loads of 2.3 g_COD_/L. Although the specific PAC load was not mentioned, the mixture of compounds such as phenol, cresol, ethylbenzene and styrene, among others, was considered the primary cause of inhibition (Seyedi et al. 2019). Similarly, a 6% load of a wastewater (89 g_COD_/L and 10.1 g_TAN_/L) from the hydrothermal liquefaction of cyanobacteria inhibited by 50% the methanogenic activity of an anaerobic sludge (Zheng et al. 2017). In this work, PAC loads completely inhibiting methanogenesis were 22.2 g_COD_/L for both the mesophilic and thermophilic conditions. The lower toxicity of the SS-PAC used in this study compared to the studies mentioned above may be due to its different charachteristics, resulting from varying pyrolysis process conditions and waste composition. Although some phenolic compounds detected in this SS-PAC are indeed toxic and inhibit microbial activity, their concentrations in the raw SS-PAC are below previously reported IC50 values for methanogenesis (Fedorak and Hrudey 1987; Blum and Speece 1991). Among the other toxic compounds present in this SS-PAC, TAN and FAN may have contributed to exacerbate the overall toxicity. TAN and FAN are well known microbial inhibitors of anaerobic digestion processes (Astals et al. 2018; Yenigün and Demirel 2013). In this work, a reduction of at least 80% of methanogenic activity was detected at SS-PAC loadings of 9.8 g_COD_/g_VSS_ with 575.6 ± 26.4 mM_TAN_ (equivalent to 10.4 ± 0.5 g_TAN_/L) under both mesophilic and thermophilic conditions. Consistent with the findings obtained here, studies examining the effects of increasing TAN concentrations on the anaerobic digestion of calcium acetate found that 9 g_TAN_/L caused a 90% reduction of methane production rates compared to control experiments with no additional TAN (Seyedi et al. 2019). Similarly, 10 g_TAN_/L (with a pH ranging between 7.4 and 7.6) inhibited approximately 90% of the specific methanogenic activity of an anaerobic granular sludge (Koster and Lettinga 1988). Another study evaluating TAN inhibition of H_2_/CO_2_ methanation reported that 7 g_TAN_/L (at a pH of about 8) inhibited methane yield by 41% and 22.3% in mesophilic and thermophilic conditions, respectively (Wang et al. 2016). Here, a higher FAN to TAN ratio (approximately 3.2 times higher), as consequence of the higher temperatures, may have contributed to the stronger inhibition observed for the thermophilic conditions compared to mesophilic ones. FAN is generally considered more toxic than the ammonium ion and FAN concentrations ranging from 0.15 up to 1.2 g_FAN_/L were recorded to be severely toxic to anaerobic digestion (Yenigün and Demirel 2013). FAN toxicity alters intracellular pH, increasing maintenance energy requirements to balance pH and depleting intracellular potassium reservoirs (Fotidis et al. 2013).

Despite having a lower COD compared to SS-PAC, PE-PAC exhibited higher toxicity. This increased toxicity was likely a consequence of its composition and concentrations of compounds. Many of the components of PE-PAC are known to be toxic to microorganisms. For instance, phenol, *o-*cresol, *m-*cresol, benzene and benzonitrile exhibited IC50 values for methanogenesis of 2.1, 0.9, 1.2 and 1.1 g/L, respectively (Blum and Speece 1991). Phenol alters membrane proteins and cell wall permeability and its toxic impacts to anaerobic digestion are well documented (Poirier et al. 2016). Cyclopentanone, at a concentration of 1 g/L, inhibited methane production from acetate by 21% (Xu, et al. 2023). Furthermore, 1,4-dioxane compromises cell membrane integrity. At 2 g/L, it reduced the specific activity of an anammox microbial consortium by 55% (Ismail et al. 2020). However, like SS-PAC, none of the organic compounds in PE-PAC was present at concentrations high enough to be considered a primary inhibitor.

Accordingly to other works, the toxicity of PAC does not derive from a specific compound but is the result of the synergistic effect of the multiple toxicants present in it. Compounds such as phenols, furans, N-heterocycles, heavy metals, TAN and FAN, when present alongside other toxic elements, can have cumulative synergistic toxic effects on microbial activity, amplifying their individual toxicities (Zhou et al. 2019; Tommaso et al. 2014). These synergistic effect impedes microbial communities' efficient degradation of compounds in the aqueous condensate and may lead to reduced process efficiencies. For instance, some N-heterocycles, such as triacetoneamine and 2-pyrrolidinone are non-toxic but can cause a synergistic cytotoxicity only when mixed (Pham et al. 2013). Additionally, operational parameters such as temperature influence the activity of microbial communities involved in the process, impacting the balance of microbial populations and their ability to metabolize organic compounds effectively (Chen et al. 2020; Schnu 2005).

### PAC components degradation and energy recovery

In addition to defining the extent of inhibition, the composition and loading of PAC greatly influenced the energy (*i.e.*, electron equivalents) recovery potential. Sewage sludge-derived PAC exhibited lower toxicity to carboxydotrophic rates than PE-PAC, however it’s components were recalcitrant to bioconversion. Conversely, despite the strong inhibition caused by PE-PAC to CO conversion rates, PE-PAC experiments showed high e-mol recoveries. Furthermore, thermophilic conditions enhanced the e-mol recovery.

The degradation of PAC components in anaerobic cultures is subject to various influencing factors, including the source of inoculum (*i.e.*, microbial composition), the molecular weight of PAC components, process operating conditions and the presence of other compounds (Chen et al. 2020; García Rea, et al. 2022). Low PAC concentrations can provide nutrients to microbial activity where many toxic components can act as substrates (Zheng et al. 2017; Chen et al. 2008). Energy recovery from SS-PAC into methane and SCCs likely resulted from the degradation of phenol, guaiacol and cresol, for instance. Conversely, nitrogen heterocyclic compounds and amides, despite being the most abundant components of SS-PAC, are considered recalcitrant to anaerobic degradation (Yang et al. 2018), thus diminishing overall energy recovery potential from this substrate (Posmanik et al. 2017). However, there are documented instances of anaerobic degradation of certain N-heterocyclic components, such as pyridine, into carboxylates and methane (Battersby and Wilson 1989; Braz et al. 2018). The efficacy of N-heterocycles removal is also affected by the presence of other compounds. For example, another work reports of pyridine degradation inhibition at phenol concentrations exceeding 400 mg/L (Sun et al. 2011). Pyridine can undergo hydroxylation, ring cleavage between C1 and C3, opening of the nitrogen ring and release of ammonium with formic and succinic acid as intermediate metabolites of its degradation. Methane was then produced directly from formic acid or from the acetate produced via succinic acid (Shi et al. 2018; Kaiser et al. 1996). Increasing TAN concentrations are considered reflection of the degree of degradation of N-heterocyclic compounds. For instance, TAN concentrations in the effluent from the anaerobic digestion of hydrothermal liquefaction wastewater of algal biomass were approximately 6 times higher compared to the influent stream and were assumed to be the result of N-heterocycles degradation (Tommaso et al. 2014). Similar results were obtained in this work, where the production of TAN in both mesophilic and thermophilic SS-PAC processes may corroborate the degradation of N-heterocycles such as pyridine. Alternatively, a part of the TAN generated here may also derive from the degradation of proteins released in the fermentation broth from cell lysis following inoculation. In previous attempts to improve the anaerobic degradability of N-heterocyclic compounds, nitrate supplementation was shown to enhance the anaerobic degradation of compounds such pyridine while serving as electron acceptor (Li et al. 2001; Shen et al. 2015). At high SS-PAC loads, concurrent nitrate removal, pyridine degradation and electron balances below 100% may suggest the occurrence of analogous mechanisms in this work, where the nitrate in SS-PAC acted as an electron acceptor and may have facilitated pyridine degradation.

In PE-PAC experiments, similarly to SS-PAC, phenol and cresol accounted for about 40% of the initial COD and their degradation possibly contributed to the higher energy recoveries. Knowledge about the anaerobic phenol and cresol degradation is already available and was reported to occur also in un-acclimatised anaerobic microbial consortia (Robazza et al. 2022; Zheng et al. 2017). For instance, removal efficiencies of cresol ranging from 10 to 60%, depending on pyrolysis temperature, were observed during biochemical methane potential studies of the aqueous phase from solid digestate at 40 °C (Hübner and Mumme 2015). One hundred milligram per litre of phenol where completely removed in 10 days during specific methanogenic activity tests at thermophilic conditions (Fang et al. 2006). In the same work, inoculum acclimation to phenol improved removal to 378 mg/L/d. The low *m-*cresol removal recorded in this work was possibly the result of the complexity of the PAC and the presence of various compounds, impeding higher removal efficacies. In an anaerobic UASB fed with a wastewater containing 900 mg/L of phenol and 320 mg/L of *m-*cresol, removal efficiencies as high as 98% and 20% were recorded for phenol and *m-*cresol, respectively, but phenol availability was considered limiting *m-*cresol removal (Zhou and Fang 1997). Vice versa, *m-*cresol highly affected phenol biodegradation in another work (Chen et al. 2016). On the other hand, in a methanogenic anaerobic continuous reactor fed with 150 mg/L phenol and with 35 mg/L of *o-* and *p-*cresol (0.25 days hydraulic retention time and a loading rate of 880 mg/L/day), a 98% removal rate was recorded for all substrates (Hajji et al. 1999). The likelihood of anaerobic degradation of other PE-PAC components such as benzene, cyclopentanone, benzonitrile, 2-chloroethanol and 1,4-dioxane seems improbable in this study. Although reports of anaerobic degradation for some exist, it is very slow, requires long adaptation times or more selected microbial consortia (Ismail, et al. 2020; Dijk et al. 2003; Vogt et al. 2011).

Reports of syngas co-fermentation with wastewaters from thermochemical processes are limited. For instance, during the continuous fermentation of syngas and increasing loadings of PAC derived from the pyrolysis of fir sawdust in a mesophilic biochar-packer bioreactor, the acclimatized microbiota recovered an average of 82% COD, yielding 45% of the COD input into carboxylates. Approximately 46% of the CO fed was consumed, but syngas accounted for only 5% of the total COD input. While the unreacted/recalcitrant COD fraction from PAC in the effluent was estimated between 25 and 52% (Küçükaǧa et al. 2022). Here, at low PACs loading, syngas was the substrate with the highest conversion rates. However, higher PAC loadings resulted in greater inhibition of syngas conversion rates compared to PAC conversion rates. The increased availability of PAC components may have favoured microorganisms capable of utilizing them as carbon and energy sources. Nevertheless, higher PAC loads inhibited degradation and energy recovery from PAC too. Similarly to the findings of this study, increasing loads (from 10 to 50%) of a post-hydrothermal liquefaction wastewater decreased the COD removal rates (from 76.8% to 36.8%) during their anaerobic digestion and were linked to the increasing toxicity and recalcitrant compounds such as N-heterocycles (Li, et al. 2022). Continuous processes allow for the acclimation of the inoculum and the enrichment of specific trophic group, leading to higher COD recoveries at higher wastewater loads. For instance, energy and carbon recoveries as high as 79% were recovered into biohythane (*i.e.*, a mixture of hydrogen gas and methane) from the anaerobic degradation of furfural, phenolics and N-heterocycles present in the post hydrothermal liquefaction wastewater of corn stalk (Si et al. 2016). Compared to other studies (Küçükaǧa et al. 2022; Li, et al. 2022; Si et al. 2016), the e-mol recovery achieved at low PAC loads in this study was relatively high for an un-acclimated inoculum and such short fermentation time. Here, syngas co-fermentation may have contributed to improve energy recovery providing extra electron donors such as CO and H_2_ to the degradation of PAC components into CH_4_, H_2_ or SCCs. Hydrogen partial pressure of 0.8 atm were reported to enhance phenol removal rates by 42% by promoting the syntrophic associations between *Syntrophorhabdus*, a phenol degrader, and methanogens during the anaerobic fermentation of phenol at high TAN concentrations (up to 8 g) (Wu et al. 2018).

Furthermore, similar to the effects of amendments such as zeolite or biochar observed during the anaerobic digestion of other PACs (Zheng et al. 2017; Kick et al. 2022), the clays and silts present in the non-volatile solid fraction of the inoculum used in this work may have enhanced process performances by improving the detoxification activity of microorganisms or even absorbed PAC compounds (Djebbar et al. 2012; Biswas et al. 2015).

## Conclusions

Recovering energy from anthropogenic and post-consumer wastes is essential to maximize resources circularity whilst minimizing environmental impacts. In this study, we proved the potential of mesophilic and thermophilic anaerobic mixed cultures to simultaneously ferment syngas and components of PACs derived from the pyrolysis of sewage sludge and mixed polyethylene plastics. However, origin and composition of the PAC influenced process performances and e-mol recovery. Comparatively, while sewage sludge-derived PAC showed lower toxicity towards carboxydotrophic activity compared to its polyethylene plastic counterpart, its components were resistant to degradation, resulting in reduced e-mol recovery. Conversely, despite its high toxicity, anaerobic mixed cultures successfully degraded some components of polyethylene plastic-derived PAC, leading to high e-mol recoveries from PAC. Products of the co-fermentations such as methane or carboxylates could be used either as energy carrier or intermediate metabolites in secondary fermentative processes, respectively. Future research should focus on the evaluation of the feasibility of the co-fermentation in continuous cultivation and on the identification of technologies that will improve the energy recovery potential and reduce PAC toxicity.

### Supplementary Information

Additional file 1: Table S1. Mass balances of the pyrolysis of sewage sludge and HDPE, LDPE plastics. Table S2. Anions and cations concentration for sewage sludge PAC and the mixed PE plastics PAC. Table S3. HPLC and GC–MS characterization of the raw aqueous condensate deriving from the fast pyrolysis of sewage sludge. The GC–MS characterization was performed by the Thunen Institute of Wood Research, (Hamburg, Germany). The total GC–MS chromatogram area: 1.06E + 08. Area of 12 identified peaks = 9.30E + 07 (88%). Area of unknown peaks = 1.27E + 07 (12%). Error bars represent standard deviation among replicates (n = 2). Table S4. HPLC and GC–MS characterization of the raw aqueous condensate deriving from the fast pyrolysis of mixed PE plastics. The GC–MS characterization was performed by the Thunen Institute of Wood Research, (Hamburg, Germany). The total GC–MS chromatogram area: 2.89E + 07. Area of 11 identified peaks = 2.22E + 07 (76.8%). Area of unknown peaks = 6.70E + 06 (23.2%). Error bars represent standard deviation among replicates (n = 2). Table S5. Conversion factors for electron balances. Figure S1. Total ammonium nitrogen (TAN), free ammonia nitrogen (FAN) and nitrate concentrations [mM] at inoculation conditions for M-SS-PAC and T-SS-PAC experiments. Error bars represent standard deviation among replicates (n = 3). Figure S2. Final average pH from fermentation at increasing sewage sludge PAC loadings. Error bars represent standard deviation among replicates (n = 3). Figure S3. e-mol recoveries from SS-PAC calculated as described in Eq. 2 for M-SS-PAC and T-SS-PAC experiments. Error bars represent standard deviation among replicates (n = 3). Figure S4. The graphs a and b show the e-equivalents balance [e-mol_products_/e-mol_syngas,fixed_] for each load of SS-PAC. Values above 100% indicate that the sum of the e-mol in the products is higher than the e-mol consumed from syngas. Error bars represent standard deviation among replicates (n = 3). Figure S5. The graphs a and b show the e-equivalents balance [e-mol_products_/e-mol_syngas,fixed]_ for each load of PE-PAC. Values above one indicate that the sum of the e-mol in the products is higher than the e-mol consumed from syngas. Error bars represent standard deviation among replicates (n = 3). Figure S6. Final average pH from fermentation at increasing mixed PE plastics PAC loadings. Error bars represent standard deviation among replicates (n = 3). Figure S7. e-mol recoveries from SS-PAC calculated as described in Eq. 2 for M-SS-PAC and T-SS-PAC experiments. Error bars represent standard deviation among replicates (n = 3).

## Data Availability

All data generated or analysed during this study are included in this published article and its Supplementary Information files.

## Abbreviations

COD: Chemical oxygen demand
eeq: Electron equivalents
e-mol: Electron moles
IC50: Inhibitory concentration of a toxicant reducing of 50% microbial activity
M-PE-PAC: Experiments evaluating the mesophilic co-fermentation of syngas and PE-PAC
M-SS-PAC: Experiments evaluating the mesophilic co-fermentation of syngas and SS-PAC
PAC: Pyrolysis aqueous condensate
PE: Polyethylene
PE-PAC: Pyrolysis aqueous condensate derived from the pyrolysis of polyethylene plastics
SCCs: Short-chain carboxylates
SS: Sewage sludge
SS-PAC: Pyrolysis aqueous condensate derived from the pyrolysis of sewage sludge
TAN: Total ammonia nitrogen
T-PE-PAC: Experiments evaluating the thermophilic co-fermentation of syngas and PE-PAC
T-SS-PAC: Experiments evaluating the thermophilic co-fermentation of syngas and SS-PAC
VSS: Volatile suspended solids
