# Supplementary material for: Energy recovery from syngas and pyrolysis wastewaters with anaerobic mixed cultures
Source: Bioresour Bioprocess. 2024 Jul 27;11(1):76. doi: 10.1186/s40643-024-00791-3 (PMC11283448; doi:10.1186/s40643-024-00791-3)
Supplement: Supplementary file 1 — Additional file 1: Table S1. Mass balances of the pyrolysis of sewage sludge and HDPE, LDPE plastics. Table S2. Anions and cations concentration for sewage sludge PAC and the mixed PE plastics PAC. Table S3. HPLC and GC–MS characterization of the raw aqueous condensate deriving from the fast pyrolysis of sewage sludge. The GC–MS characterization was performed by the Thunen Institute of Wood Research, (Hamburg, Germany). The total GC–MS chromatogram area: 1.06E + 08. Area of 12 identified peaks = 9.30E + 07 (88%). Area of unknown peaks = 1.27E + 07 (12%). Error bars represent standard deviation among replicates (n = 2). Table S4. HPLC and GC–MS characterization of the raw aqueous condensate deriving from the fast pyrolysis of mixed PE plastics. The GC–MS characterization was performed by the Thunen Institute of Wood Research, (Hamburg, Germany). The total GC–MS chromatogram area: 2.89E + 07. Area of 11 identified peaks = 2.22E + 07 (76.8%). Area of unknown peaks = 6.70E + 06 (23.2%). Error bars represent standard deviation among replicates (n = 2). Table S5. Conversion factors for electron balances. Figure S1. Total ammonium nitrogen (TAN), free ammonia nitrogen (FAN) and nitrate concentrations [mM] at inoculation conditions for M-SS-PAC and T-SS-PAC experiments. Error bars represent standard deviation among replicates (n = 3). Figure S2. Final average pH from fermentation at increasing sewage sludge PAC loadings. Error bars represent standard deviation among replicates (n = 3). Figure S3. e-mol recoveries from SS-PAC calculated as described in Eq. 2 for M-SS-PAC and T-SS-PAC experiments. Error bars represent standard deviation among replicates (n = 3). Figure S4. The graphs a and b show the e-equivalents balance [e-molproducts/e-molsyngas,fixed] for each load of SS-PAC. Values above 100% indicate that the sum of the e-mol in the products is higher than the e-mol consumed from syngas. Error bars represent standard deviation among replicates (n = 3). Figure S5. The gr [file 40643_2024_791_MOESM1_ESM.docx]

Energy Recovery from Syngas and Pyrolysis Wastewaters during Anaerobic Mixed Culture Fermentations

Alberto Robazza^1^, Anke Neumann^1^

^1^ Institute of Process Engineering in Life Sciences 2: Electro Biotechnology, Karlsruhe Institute of Technology - KIT, 76131 Karlsruhe, Germany;

# 1 Mass balance of the pyrolysis of sewage sludge and mixed PE plastics

Table S1. Mass balances of the pyrolysis of sewage sludge and HDPE, LDPE plastics

| Sample |  | Dry SS | SS | SS Av. | HDPE | LDPE | Av. PE |
| --- | --- | --- | --- | --- | --- | --- | --- |
| Waste Flow | g/h | 1200 | 1200 | 1200 | 1000 | 1000 | 1000 |
| PAC | g/h | 155.8 | 371.8 | 263.8 | 66.8 | 55.2 | 61 |
| Syngas | g/h | 236.2 | 241 | 238.6 | 266.4 | 250.2 | 258.3 |
| Yield_PAC+Syngas_ | % | 32.7 | 51.1 | 41.9 | 33.3 | 30.5 | 31.9 |

# 2 Characterization of the sewage sludge PAC and the mixed PE plastics PAC

Table S2. Anions and cations concentration for sewage sludge PAC and the mixed PE plastics PAC.

| Parameter | Concentration | Sewage Sludge PAC | Mixed PE Plastics PAC |
| --- | --- | --- | --- |
| Fluoride | mg/l | n.b. | n.b. |
| Chloride | mg/l | < 100 | 1720 |
| Nitrite | mg/l | < 5.0 | n.b. |
| Bromide | mg/l | n.b. | < 5.0 |
| Nitrate | mg/l | 448 | 5.87 |
| Phosphate | mg/l | < 10 | < 10 |
| Sulfate | mg/l | 1940 | 168 |
| Aluminium | mg/l | 1.8 | 1.6 |
| Arsenic | mg/l | 3.7 | < 0.1 |
| Barium | mg/l | 0.11 | < 0.1 |
| Calcium | mg/l | 15 | 6.2 |
| Cadmium | mg/l | < 0.1 | < 0.1 |
| Cobalt | mg/l | 0.22 | < 0.1 |
| Chrome | mg/l | 5.9 | 3.7 |
| Copper | mg/l | < 0.1 | < 0.1 |
| Iron | mg/l | 47 | 26 |
| Magnesium | mg/l | 1.6 | 0.23 |
| Manganese | mg/l | < 0,1 | 0.32 |
| Nickel | mg/l | 13 | 3.3 |
| Phosphor | mg/l | 4.3 | 0.12 |
| Lead | mg/l | < 0.1 | < 0.1 |
| Antimony | mg/l | 3.7 | < 0.2 |
| Silicium | mg/l | 128 | 29 |
| Tin | mg/l | 0.67 | 0.63 |
| Thallium | mg/l | < 0.1 | < 0.1 |
| Zinc | mg/l | 0.11 | 1.2 |

Table S3. HPLC and GC-MS characterization of the raw aqueous condensate deriving from the fast pyrolysis of sewage sludge. The GC-MS characterization was performed by the Thunen Institute of Wood Research, (Hamburg, Germany). The total GC-MS chromatogram area: 1.06E+08. Area of 12 identified peaks = 9.30E+07 (88%). Area of unknown peaks = 1.27E+07 (12%). Error bars represent standard deviation among replicates (n=2).

| Compound |  | Concentration g/L | | |
| --- | --- | --- | --- | --- |
|  |  | Average | St.Dev. |  |
| Acetate | HPLC | 9.9 | 0.02 |  |
| Propionate | HPLC | 3.73 | 0.05 |  |
| Butyrate | HPLC | 1.85 | 0.01 |  |
| Phenol | HPLC | 0.53 | 0.02 |  |
| Guaiacol | HPLC | 0.62 | 0.01 |  |
| *p*-Cresol | HPLC | 0.49 | 0.02 |  |
| *m*-Cresol | HPLC | 3.46 | 0.24 |  |
| Pyridine | HPLC | 3.1 | 0.02 |  |
| 4-Penten-2-one, 4-methyl- (NIST MQ 92) | GC-MS | 2.46 | 0.12 |  |
| 3-Penten-2-one, 4-methyl- (NIST MQ 92) | GC-MS | 5.69 | 0.22 |  |
| 2-Pentanone, 4-amino-4-methyl- (NIST MQ 86) | GC-MS | 27.69 | 0.42 |  |
| Acetamide (NIST MQ 94) | GC-MS | 1.18 | 0.1 |  |
| unknown N-Compound (no NIST spectrum found) | GC-MS | 41.11 | 0.93 |  |
| unknown N-Compound (no NIST spectrum found) | GC-MS | 1.06 | 0.03 |  |
| unknown N-Compound (no NIST spectrum found) | GC-MS | 1.38 | 0.02 |  |
| Triacetoneamine (NIST MQ 87) | GC-MS | 32.55 | 0.01 |  |
| 2-Pyrrolidinone (NIST MQ 94) | GC-MS | 1.59 | 0.03 |  |
| unknown N-Compound (no NIST spectrum found) | GC-MS | 0.60 | 0.02 |  |

Table S4. HPLC and GC-MS characterization of the raw aqueous condensate deriving from the fast pyrolysis of mixed PE plastics. The GC-MS characterization was performed by the Thunen Institute of Wood Research, (Hamburg, Germany). The total GC-MS chromatogram area: 2.89E+07. Area of 11 identified peaks = 2.22E+07 (76.8%). Area of unknown peaks = 6.70E+06 (23.2%). Error bars represent standard deviation among replicates (n=2).

| Compound |  | | | | Concentration g/L | | | |
| --- | --- | --- | --- | --- | --- | --- | --- | --- |
|  |  | | Average | | | | St.Dev. | |
| Acetate | | HPLC | | 14.26 | | 0.26 | |  |
| Propionate | | HPLC | | 2.73 | | 0.01 | |  |
| Butyrate | | HPLC  LC | | 0.62 | | 0.07 | |  |
| *p*-Cresol | | HPLC | | 0.37 | | 0.02 | |  |
| Benzene | | HPLC | | 0.58 | | 0.03 | |  |
| Butanol, 1- | | GC-MS | | 0.39 | | 0.01 | |  |
| Acetol (Hydroxyacetone) | | GC-MS | | 8.93 | | 0.21 | |  |
| Cyclopentanone | | GC-MS | | 1.21 | | 0.24 | |  |
| Phenol | | GC-MS | | 9.62 | | 0.08 | |  |
| *m*-Cresol | | GC-MS | | 1.24 | | 0.12 | |  |
| Benzonitrile (NIST MQ 84) | | GC-MS | | 1.14 | | 0.01 | |  |
| 1,3-Dioxolane, 2,2-dimethyl- | | GC-MS | | 0.20 | | 0.02 | |  |
| 1,4-Dioxane (NIST MQ 83) | | GC-MS | | 0.16 | | 0.01 | |  |
| 2-Chloroethanol (NIST MQ 86) | | GC-MS | | 0.55 | | 0.004 | |  |

# 3 Conversion factors for electron balancing

| Compound | Chemical Formula | Molecular Weight | mol e^-^ /mol |
| --- | --- | --- | --- |
| Formate | CH_2_O_2_ | 46.1 | 2.0 |
| Acetate | C_2_H_4_O_2_ | 60.0 | 8.0 |
| Propionate | C_3_H_6_O_2_ | 74.0 | 14.0 |
| Butyrate | C_4_H_8_O_2_ | 88.1 | 20 |
| Hydrogen | H_2_ | 2.0 | 2.0 |
| Carbon Monoxide | CO | 28.0 | 2.0 |
| Carbon Dioxide | CO_2_ | 44.0 | 0.0 |
| Methane | CH_4_ | 16.0 | 8.0 |

Table S5. Conversion factors for electron balances.

# 4 Syngas and Sewage Sludge PAC co-fermentation


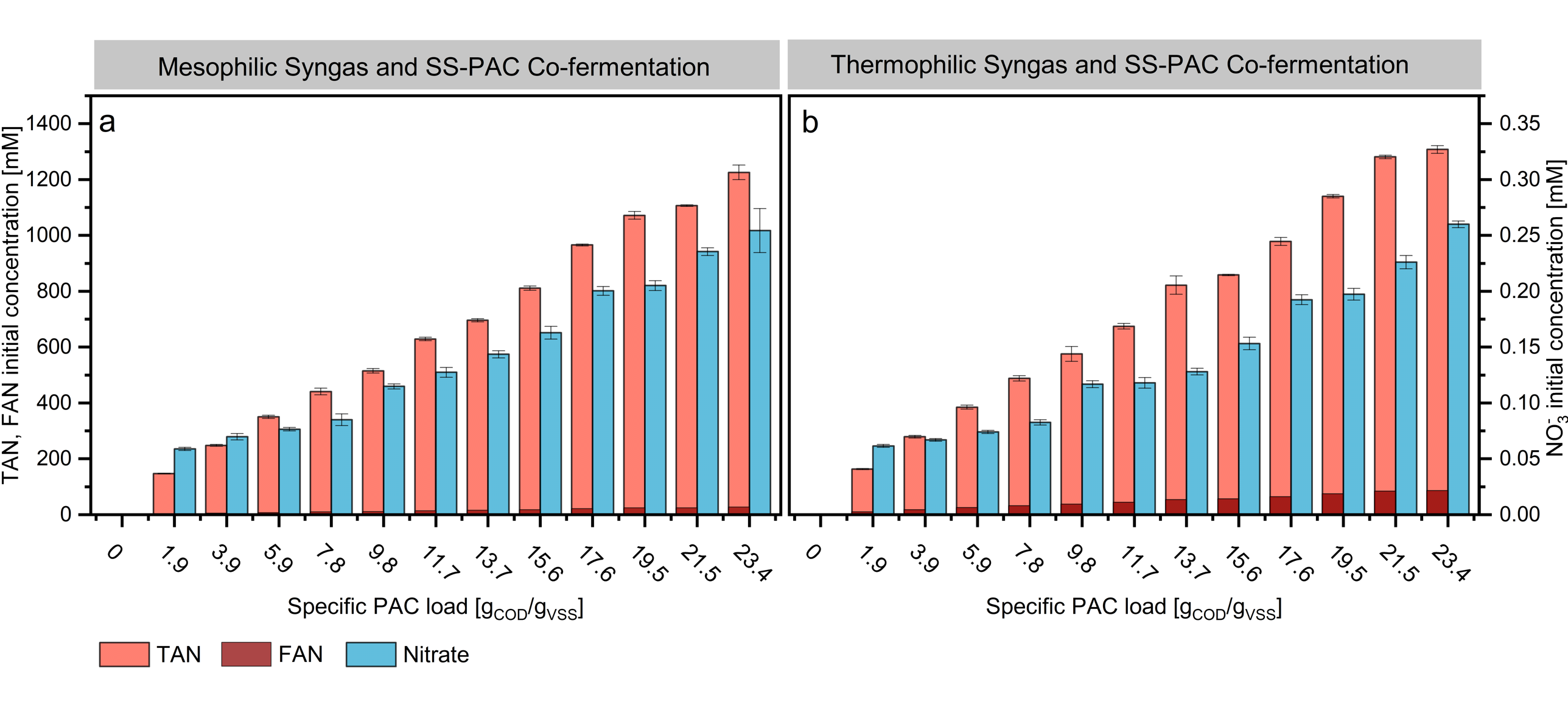
 Figure S1. Total ammonium nitrogen (TAN), free ammonia nitrogen (FAN), and nitrate concentrations [mM] at inoculation conditions for M-SS-PAC and T-SS-PAC experiments. Error bars represent standard deviation among replicates (*n*=3).



 Figure S2. Final average pH from fermentation at increasing sewage sludge PAC loadings. Error bars represent standard deviation among replicates (n=3).


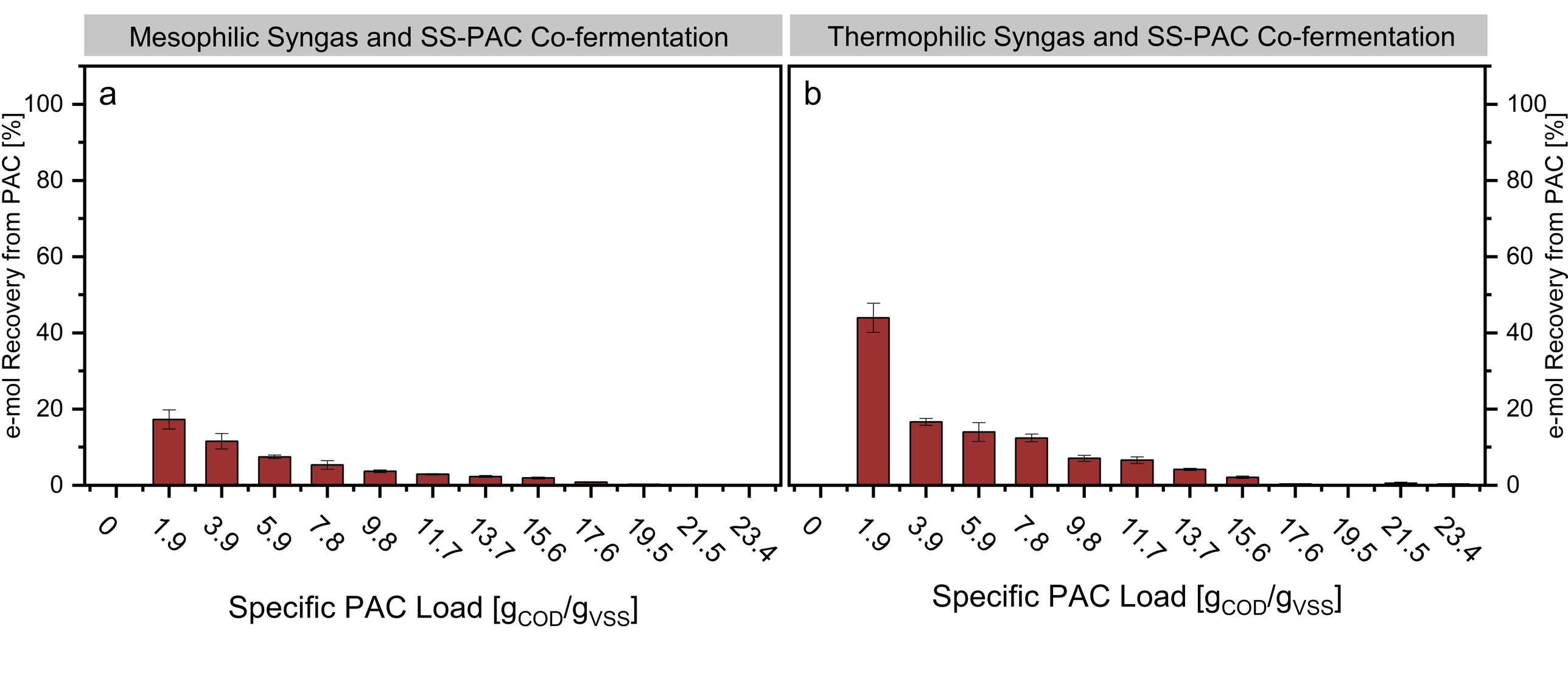


Figure S3. e-mol recoveries from SS-PAC calculated as described in Eq.2 for M-SS-PAC and T-SS-PAC experiments. Error bars represent standard deviation among replicates (n=3).



 Figure S4. The graphs a and b show the e-equivalents balance [e-mol_products_/e-mol_syngas,fixed_] for each load of SS-PAC. Values above 100% indicate that the sum of the e-mol in the products is higher than the e-mol consumed from syngas. Error bars represent standard deviation among replicates (*n*=3).

# 5 Syngas and Polyethylene Plastics PAC co-fermentation

#

 Figure S5. The graphs a and b show the e-equivalents balance [e-mol_products_/e-mol_syngas,fixed_] for each load of PE-PAC. Values above one indicate that the sum of the e-mol in the products is higher than the e-mol consumed from syngas. Error bars represent standard deviation among replicates (n=3).


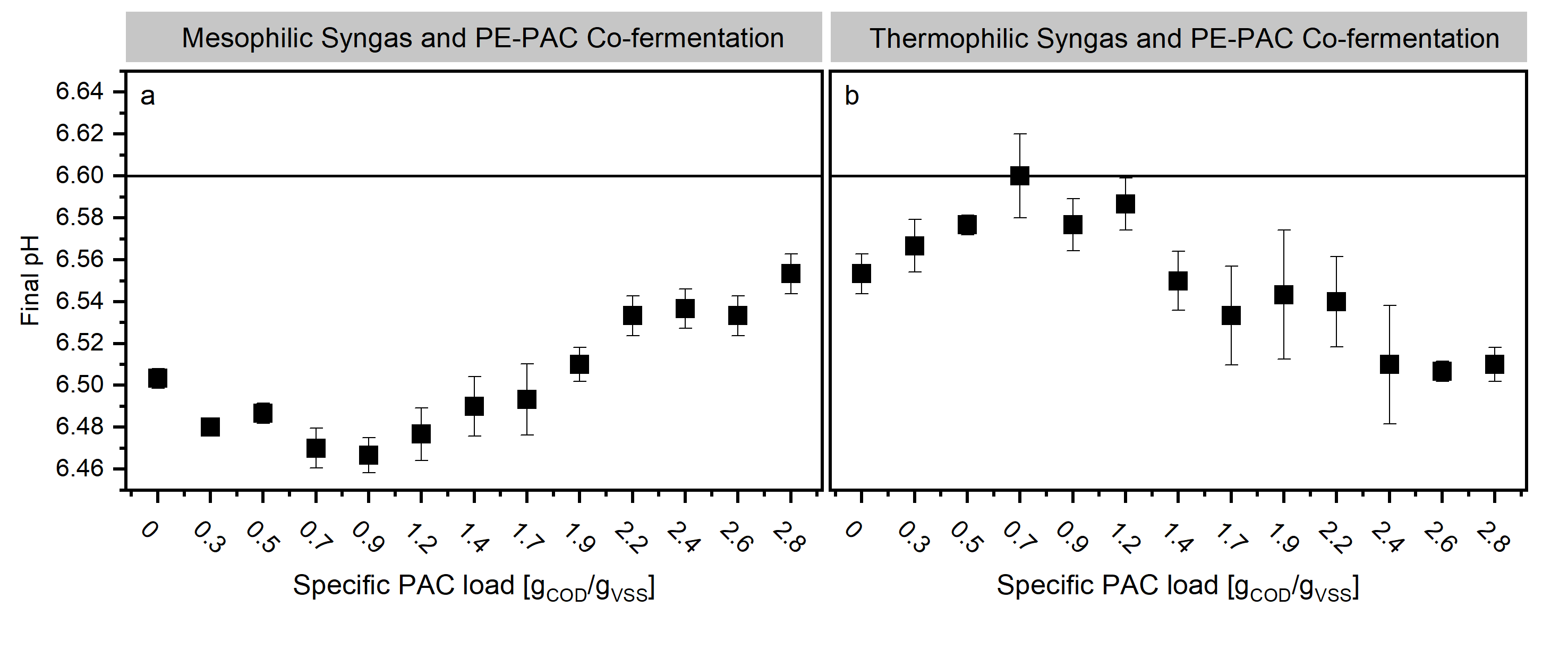
 Figure S6. Final average pH from fermentation at increasing mixed PE plastics PAC loadings. Error bars represent standard deviation among replicates (n=3).



 Figure S7. e-mol recoveries from SS-PAC calculated as described in Eq.2 for M-SS-PAC and T-SS-PAC experiments. Error bars represent standard deviation among replicates (n=3).
